# The mylohyoid line is highly variable but does not affect the microarchitecture of the edentulous alveolar bone – an anatomical micro-CT study

**DOI:** 10.1186/s12903-024-04293-8

**Published:** 2024-05-03

**Authors:** Danijel Domic, Julia Kappenberger, Kristina Bertl, Lena Hirtler, Patrick Heimel, Christian Ulm

**Affiliations:** 1 Division of Oral Surgery, University Clinic of Dentistry, Medical University of Vienna, Sensengasse 2a, Vienna, 1090 Austria; 2 Department of Periodontology, Dental Clinic, Faculty of Medicine, Sigmund Freud University, Freudplatz 3, Vienna, 1020 Austria; 3 Department of Periodontology, Blekinge Hospital, Byggnad 13, Hälsovägen, Karlskrona, 371 41 Sweden; 4 Center for Anatomy and Cell Biology, Medical University of Vienna, Währinger Strasse 13, Vienna, 1090 Austria; 5 Karl Donath Laboratory for Hard Tissue Histology and Bone Regeneration, University Clinic of Dentistry, Medical University of Vienna, Sensengasse 2a, Vienna, 1090 Austria; 6 Ludwig Boltzmann Institute for Traumatology, The research centre in cooperation with AUVA, Donaueschingenstrasse 13, Vienna, 1200 Austria; 7 Austrian Cluster for Tissue Regeneration, Donaueschingenstrasse 13, Vienna, 1200 Austria

**Keywords:** Mylohyoid line, Bone quality, Mandible, Micro-computed tomography

## Abstract

**Objectives:**

To evaluate in the absence of teeth the variability of the mylohyoid line (ML), the microarchitecture of the adjacent bone, and whether the variable prominence/width of the ML is associated with the quality of the adjacent bone.

**Methods:**

µCT scans of 28 human mandibles from anatomical specimens were analyzed. The following parameters were assessed in four edentulous areas (first and second premolar (PM), first, second, and third molar (M1/2/3)): ML width, cortical thickness (CtTh), average cortical- (Avg.Ct.BV/TV), and trabecular bone volume fraction (Avg.Tb.BV/TV).

**Results:**

The ML width increased from the PM towards the M2 region, which also showed the highest variance (range: 0.4–10.2 mm). The CtTh showed a decrease in the M3 region, while Avg.Ct.BV/TV and Avg.Tb.BV/TV hardly differed among the regions. In the multivariable model on the effect of the various parameters on the ML width, only gender and tooth region were significant. Specifically, male specimens were associated with a wider ML width compared to female specimens and the M2 region was associated with a wider ML width compared to the other tooth regions.

**Conclusion:**

The ML width was not associated with the cortical and trabecular bone quality in the adjacent bone, while gender and tooth region had a significant effect. Specifically, the ML width was lower in female, but peaked in the M2 region with a median width of 3–4 mm.

**Clinical relevance:**

From a clinical point of view, it was confirmed that the ML is in general a highly variable structure, especially in the M2 region, but the ML width does not allow any conclusions on the bone quality. Altogether, this underlines the need for an individual and accurate diagnostic prior to any surgical intervention.

**Supplementary Information:**

The online version contains supplementary material available at 10.1186/s12903-024-04293-8.

## Introduction

Tooth loss in the posterior mandible is often associated with numerous challenges, such as reduction of chewing efficacy, alveolar ridge resorption, elongation of the opposing teeth, and tilting of the neighboring teeth leading to occlusal interferences, etc [1, 2]. To avoid these consequences of tooth loss dental implants are a well-established method with a high survival rate of 96–98% after 10 years [3, 4]. However, one potential complication during implant installation in the posterior mandible is perforation of the lingual cortex, i.e., lingual plate perforation (LPP). LPP can cause life-threatening bleeding due to injury of the peri-mandibular vessels followed by compression of the airways [5, 6]; the risk for such severe complication depends on many factors such as the distance of the blood vessels from the mylohyoid line [7]. The risk for LPP per se varies in different reports and tooth regions between 1 and 31% [6, 8]. For example, the lowest risk was reported in the region of the second premolar (PM; 7%) followed by the region of the first molar (M1; 9%), and finally the second molar (M2) with a 3- to 4-times higher risk (31%) [8]. Hence, to avoid LPP presurgical assessment of the posterior mandible by clinical examination and if indicated three-dimensional imaging, such as cone-beam computed tomography (CBCT), is recommended.

The mylohyoid line (ML) lies within this area of the mandible and forms the lingual balcony/undercut. It runs from the apical aspect of the central suture of the mandible diagonally in a dorso-cranial direction, becomes more prominent in the posterior region and ends below the retromolar trigonum [9]. The ML represents the upper border of the submandibular fossa and an anchorage site for the mylohyoid muscles. However, its shape and dimension vary considerably among different patients [10], which raises the question whether its bony architecture (i.e., cortical bone thickness and quality, trabecular bone density, etc.) also varies between patients and/or depending on its shape and dimension.

The cortical thickness (CtTh) and structure/quality of other regions relevant for dental implant installation, such as buccal and palatal/lingual aspects of the anterior and posterior mandible and maxilla, and the maxillary sinus floor have previously been investigated in anatomical specimens by micro-CT (µCT), histomorphometry, and/or CBCT [11–15]. For example, the CtTh of the posterior mandibular lingual plate has been described to vary between 1.0 mm in the region of the third molar (M3; measured by CBCT) up to 2.4 mm in the PM region of severely atrophic edentulous mandibles (measured by histomorphometry) [14, 15]. However, specific information on the CtTh and quality of underlying trabecular bone of the ML is missing in the literature. As increased knowledge on the shape, dimension, and bone quality of the ML might help to reduce the risk of LPP, the present study aimed to evaluate in the absence of teeth the variability of the ML, the microarchitecture of the adjacent bone, and whether the variable prominence/width of the ML is associated with the quality of the adjacent bone.

## Materials and methods

### Selection and preparation of the specimen

The present project was approved by the ethics committee of the Medical University of Vienna (EK-Nr: 1933/2020). Human anatomical specimens embalmed in formaldehyde-phenol solution were collected from body donations bequeathed to the Anatomical Institute of the Medical University of Vienna for medical-scientific research and training purposes according to the following inclusion criteria: (1) partially or fully edentulous posterior mandible; (2) no visible bone defects/pathologies; and (3) no dental implants. The preparation of the whole bodies was previously performed by perfusion of the vascular system with a phenol-formaldehyde solution (4% phenol and 1% formalin in water) for a minimum of 24 h, after which the whole corpses were kept in the same solution for at least 6 months. Per protocol 30 hemi-mandibles of 30 specimen were included. All samples were trimmed to the region from mesial of the mental foramen to above the trigonum retromolare.

### µCT scanning

Samples were tightly packed in standardized containers and scanned by µCT (VISCOM X8060 NDT; transmission tube, digital detector, voxel size 31.75 µm^3^, 130 kV, 320 µA; diamond filter with 0.75 mm copper; exposition time: 1400 ms; zoom factor: 4000; 1440 projections; scanning time ca. 34 min).

### Image orientation and definition of the region of interest (ROI)

**Fig. 1:**
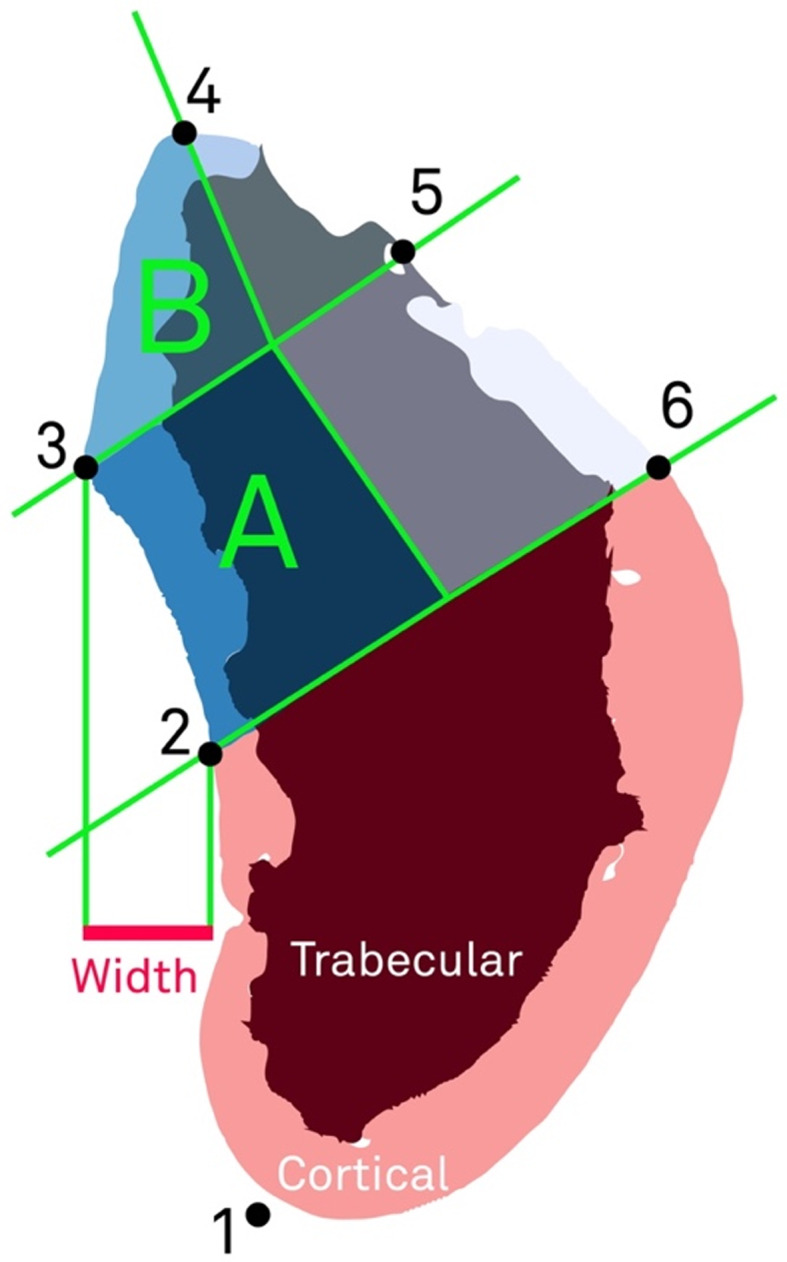
Illustration of the regions of interest

The µCT images were reconstructed and imported into an image software (Fiji) [16], in which the whole specimen/volume was vertically oriented, with the ML positioned to the left side of the screen. The vertical orientation was based on the PM region, which allowed to achieve a position resembling well the natural position of the mandible. On the cross-sections, six points were marked to define the ROI (Fig. 1):

1. At the most inferior depth of the mandible along a straight line from point 2 parallel to the inferior part of the lingual bone plate;
2. The inferior part of the lingual bone plate forms a relatively straight section. In the superior direction, the bone bends gradually in the lingual direction, forming the ML. Point 2 is placed on the most superior point along the straight section of the lingual bone plate at the point right before the bone begins to bend in the lingual direction towards point 3;
3. The most prominent lingual aspect of the ML;
4. At the lingual edge of the alveolar process;
5. At the buccal edge of the alveolar process;
6. Buccal of the alveolar process, approximately in the same distance to point 5, as between point 2 and 3.

These points were placed by a single examiner on approximately 13 slices in the µCT stack and linearly interpolated between the remaining slices.

The width of the ML was defined as the distance between point 2 and the most prominent lingual aspect of the ML (point 3). Accordingly, in the extension of the most prominent point of the ML to the buccal, the mandible was divided into a sub- (A) and supra- (B) mylohyoid region. Region B is defined as an isosceles triangle with a height of 4 mm and the line between (3) and (4) forming the base. Region A is a quadrilateral composed of the line between (2) and (3), the adjacent edge of region B, and a line parallel to the adjacent edge of region B running through (2) and (6).

All cross-sectional edentulous images were allocated to the corresponding tooth site: PM (including first and second PM), M1, M2, and M3. In partially edentulous specimens, the remaining teeth were used as reference, whereas in fully edentulous specimens, the mental foramen and standard crown width was used [17, 18].

Cortical and trabecular regions were separated with the following procedure on a binary mask of the bone. To remove pores in the cortical bone, a closing operation (iterations 2, count 3) was applied to a copy of the binary mask down-sampled to 25%. The closed image was converted to a thickness map, and all structures, which were further away from the outer bone surface than the local thickness, were removed. This left only a thick layer of bone on the outer edge of the mandible, i.e., the cortical bone. To apply the separated cortex to the full resolution, the image was upscaled to the full resolution, dilated (iterations 2, count 3) and the image calculator applied with the minimum operation relative to the original segmented mask followed by another closing operation (iterations 8, count 3). The resulting image was a binary mask, which only contained the cortical bone.

### Quantitative measurements of the cortical and trabecular bone micro-architecture

Within the ROI (i.e., region A and B) the following parameters were analyzed:

- CtTh (mm).
- Average cortical bone volume fraction (Avg.Ct.BV/TV; %).
- Average trabecular bone volume fraction (Avg.Tb.BV/TV; %).

### Statistical analysis

Statistical analysis was performed using STATA 17.0 for Mac, and *p*-values ≤ 0.05 were considered as statistically significant. Non-normal distribution of the data was confirmed by the Shapiro-Wilk test and the median, first (Q1) and third quartile (Q3), and minimum and maximum values were calculated for all continuous parameters. Correlations among the parameters were analyzed by Spearman’s correlation coefficient. The comparison between the different tooth regions for the different parameters was performed by Friedman test, and in case of significance the Wilcoxon-signed-rank test was applied for pairwise comparison. The main analysis, which also took into account that specimens contributed with different tooth regions, was focusing on the primary outcome parameter “ML width” (continuous parameter). A generalized linear model [Generalized estimating equations (GEE) population – averaged model) was used in 2 steps using the bootstrap method with 500 replications. In a first step, the following predictors were tested one by one in univariable analyses: (1) gender (female / male), (2) tooth region (PM / M1 / M2 / M3), and each of the bone quality parameters, i.e., (3) CtTh, (4) Avg.Ct.BV/TV, and (5) Avg.Tb.BV/TV. In the second step, all predictors with a *p*-value ≤ 0.02 in the univariable analysis were combined in a multivariable analysis. In addition, a pairwise comparison was performed across the levels of the predictor “tooth region” (i.e., M1 vs. PM, M1 vs. M2, M1 vs. M3, etc.).

## Results

Sixty-six edentulous regions (PM: 9 samples, M1: 23 samples, M2: 14 samples, M3: 20 samples) of 28 specimens were included in the final evaluation (15 male / 13 female). The age of 22 specimens varied from 67 to 89 years (mean age 78.1 years), whereas for 6 specimens, detailed information on age was not available but assumable > 60 years of age. The reason for exclusion of 2 specimens were bone defects, which became apparent only after µCT scanning. Further, all dentate regions and all regions with the presence of foreign bodies or any pathologies (e.g., bone cysts, etc.) were also excluded.

### Comparison of the ML width and bone microarchitecture among the tooth regions

The median ML width increased from the PM region towards the region of M2 and then decreased again in the region of M3. A significant difference in ML width was observed between the PM and M1 region (*p* = 0.02), and between the M2 and M3 region (*p* = 0.05).

**Fig. 2:**
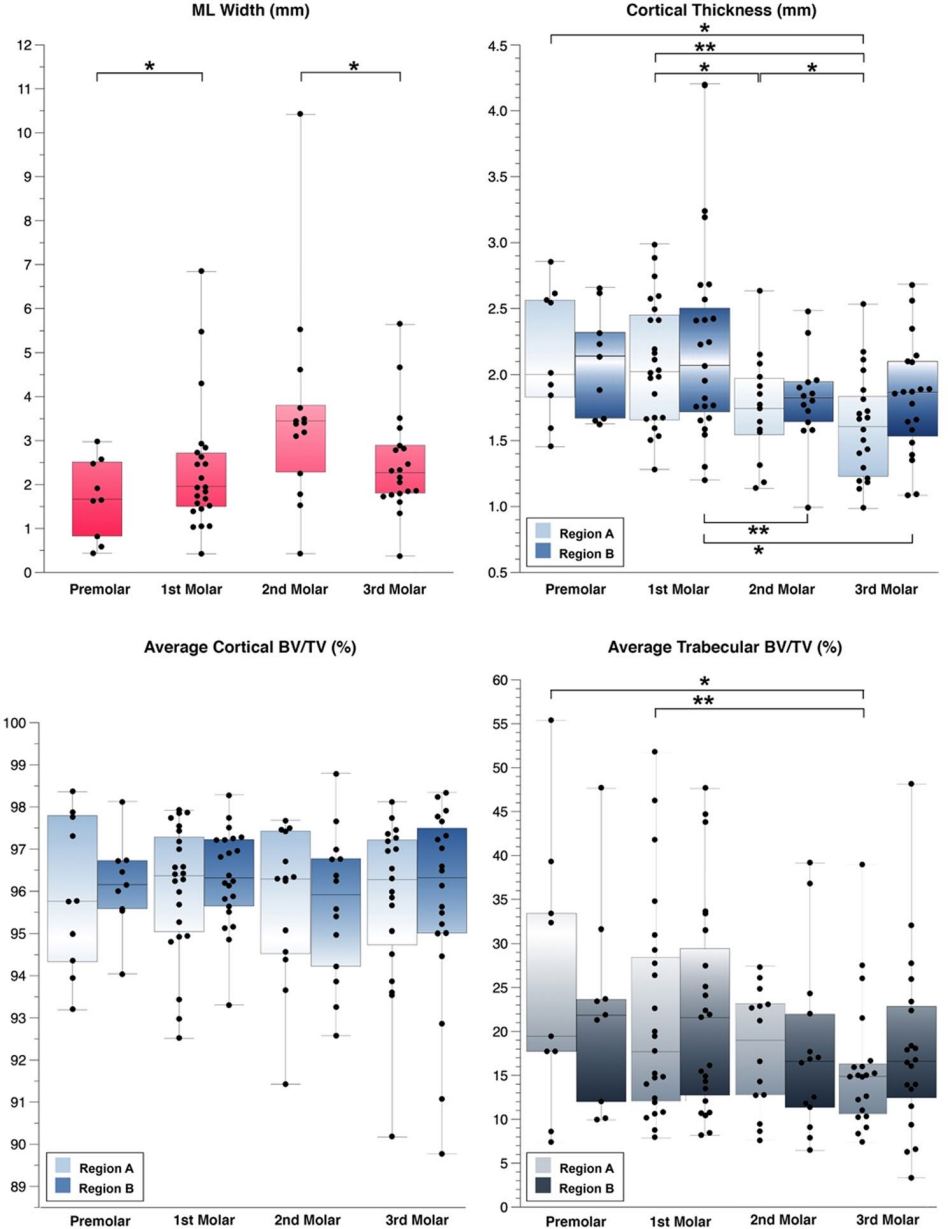
The boxplots represent the mylohyoid line width (ML Width), cortical thickness, average cortical bone volume fraction (BV/TV), and average trabecular BV/TV of each tooth region, i.e., premolar, first, second, and third molar, as well as of the sub- (Region A) and supra- (Region B) mylohyoid region. The horizontal lines indicate the significant results of the pairwise analyses (* for *p* ≤ 0.05, ** for *p* ≤ 0.01)

The CtTh of region A was comparable in the PM and M1 region, but slightly decreased towards M3. A significantly higher CtTh was found in the region of PM (*p* = 0.02), M1 (*p* < 0.01), and M2 (*p* = 0.03) in comparison to the M3 region; moreover, the M1 region showed a significantly higher CtTh than M2 (*p* = 0.01). The highest median CtTh of region B was found in PM region, but decreased towards M3 region. A significantly higher CtTh was found in the M1 region in comparison to M2 (*p* < 0.01) and M3 (*p* = 0.05) region. The Avg.Ct.BV/TV differed neither in region A nor B among the 4 tooth regions. Avg.Tb.BV/TV of region A slightly decreased from PM towards M3 region. A significantly higher Avg.Tb.BV/TV was found in the PM (*p* = 0.02) and M1 region (*p* < 0.01) in comparison to the M3 region. In region B the Avg.Tb.BV/TV did not differ significantly across the 4 tooth regions. The values of ML width, CtTh, Avg.Ct.BV/TV, and Avg.Tb.BV/TV of the 4 tooth regions, as well as the significant results of pairwise analysis are presented in Fig. 2.

### Correlations between the bone parameters

The results of the Spearman’s correlations are presented in Table 1. A significant positive correlation was observed for CtTh between region A and B in the PM (Spearman-Coef.: 0.78, *p* = 0.01), M1 (Spearman-Coef.: 0.78, *p* < 0.01), and M3 (Spearman-Coef.: 0.69, *p* < 0.01) region. Further, a significant positive correlation was observed for Avg.Ct.BV/TV between region A and B in the PM (Spearman-Coef.: 0.68, *p* = 0.04) and M3 (Spearman-Coef.: 0.62, *p* < 0.01) region.

**Table 1:** Correlations of (I) the cortical parameters between the sub- (A) and supramylohyoid (B) region, (II) between ML width and the bone microarchitecture parameters in region A, and (III) between the bone microarchitecture parameters in region A

		PM	M1	M2	M3		
**I) Correlations of the cortical parameters between region A and B**							
CtTh in region A vs. B	Spearman-Coef.	**0.78**	**0.78**	0.46	**0.69**		
	*p*-value	**0.01**	**< 0.01**	0.09	**< 0.01**		
Avg.Ct.BV/TV in region A vs. B	Spearman-Coef.	**0.68**	0.21	0.52	**0.62**		
	*p*-value	**0.04**	0.34	0.06	**< 0.01**		
**II) Correlations between ML width and bone parameters in region A**							
ML width vs. CtTh	Spearman-Coef.	-0.47	-0.04	-0.17	0.23		
	*p*-value	0.21	0.85	0.55	0.32		
ML width vs. Avg.Ct.BV/TV	Spearman-Coef.	0.20	-0.18	**-0.55**	0.03		
	*p*-value	0.61	0.40	**0.04**	0.89		
ML width vs. Avg.Tb.BV/TV	Spearman-Coef.	-0.32	0.07	0.44	-0.11		
	*p*-value	0.41	0.77	0.11	0.65		
**III) Correlations between bone parameters in region A**							
Avg.Ct.BV/TV vs. CtTh	Spearman-Coef.	-0.53	-0.04	-0.10	0.06		
	*p*-value	0.14	0.84	0.73	0.80		
Avg.Tb.BV/TV vs. CtTh	Spearman-Coef.	**0.85**	**0.67**	0.48	0.33		
	*p*-value	**< 0.01**	**< 0.01**	0.08	0.15		
Avg.Tb.BV/TV vs. Avg.Ct.BV/TV	Spearman-Coef.	-0.62	-0.32	**-0.62**	**-0.59**		
	*p*-value	0.08	0.14	**0.02**	**< 0.01**		

A- submylohyoid region; Avg.Ct.BV/TV- average cortical bone volume fraction; Avg.Tb.BV/TV- average trabecular bone volume fraction; B- supramylohyoid region; CtTh- cortical thickness; M1/2/3- first/second/third molar region; ML- mylohyoid line; PM- premolar region; Spearman-Coef.- Spearman coefficient.

Within region A, ML width correlated significantly only with one of the parameters and only in the M2 region. Specifically, a significant negative correlation was observed between ML width and Avg.Ct.BV/TV in M2 region (Spearman-Coef.: -0.47, *p* = 0.04).

Within region A, no significant correlations were observed between Avg.Ct.BV/TV and CtTh in any of the 4 tooth regions. Avg.Tb.BV/TV correlated significantly positive with CtTh in the PM (Spearman-Coef.: 0.85, *p* < 0.01) and M1 (Spearman-Coef.: 0.67, *p* < 0.01) region, while a significant negative correlation was observed between Avg.Ct.BV/TV and Avg.Tb.BV/TV in the M2 (Spearman-Coef.: -0.62, *p* = 0.02) and M3 (Spearman-Co: -0.59, *p* < 0.01) region.

### Effect of gender, tooth region, and bone microarchitecture on ML width

In the univariable models, the possible effect of gender, tooth region, and bone microarchitecture on the ML width was assessed. Gender and tooth region had a significant effect on the ML width and were combined in the final/multivariable model. A significant effect was confirmed for both parameters. Specifically, samples from male specimens were associated with a wider ML width compared to samples from female specimens [observed coefficient (OC): 0.50, 95% CI: 0.05–0.94, *p* = 0.03]. Further, all molar regions tended to be associated with a wider ML width compared to the PM region with the M2 region being significant (OC: 1.57, 95% CI: 0.67–2.48, *p* < 0.01). The additional pairwise comparison among the 4 tooth regions confirmed, that the M2 region was associated with a significant wider ML width compared to all 3 other tooth regions (i.e., PM, M1, and M3 region). The results are presented in detail in Table 2.

**Table 2:** Results of the generalized linear models for the primary outcome parameter ML width; (a) univariable analyses; (b) multivariable analysis; (c) pairwise comparison between the tooth regions. A positive coefficient (OC) indicates a wider ML width

Parameter	OC	95% CI		*p*-value	
		Lower	Upper		
**Univariable analyses**					
**Gender**					
Female	Base				
Male	**0.59**	**0.11**	**1.07**	**0.02**	
**Tooth region**					
PM	Base				
M1	0.64	-0.06	1.34	0.07	
M2	**1.59**	**0.75**	**2.43**	**< 0.01**	
M3	0.75	-0.04	1.54	0.06	
CtTh	-0.43	-1.16	0.30	0.25	
Avg.Ct.BV/TV	-13.69	-48.07	20.69	0.44	
Avg.Tb.BV/TV	-0.96	-4.34	2.43	0.58	
**Multivariable analysis**					
**Gender**					
Female	Base				
Male	**0.50**	**0.05**	**0.94**	**0.03**	
**Tooth region**					
PM	Base				
M1	0.64	-0.11	1.39	0.09	
M2	**1.57**	**0.67**	**2.48**	**< 0.01**	
M3	0.75	-0.08	1.58	0.08	
**Pairwise comparison between the tooth regions**					
M1 vs. PM	0.64	-0.09	1.38	0.09	
M2 vs. PM	**1.57**	**0.70**	**2.45**	**< 0.01**	
M3 vs. PM	0.75	-0.10	1.60	0.08	
M2 vs. M1	**0.93**	**0.15**	**1.71**	**0.02**	
M3 vs. M1	0.11	-0.55	0.76	0.75	
M3 vs. M2	**-0.82**	**-1.62**	**-0.03**	**0.04**	

Bold values indicate statistical significance

Avg.Ct.BV/TV- average cortical bone volume fraction; Avg.Tb.BV/TV- average trabecular bone volume fraction; CI- confidence interval; CtTh- cortical thickness; M1/2/3- first/second/third molar region; OC- observed coefficient; PM- premolar region

## Discussion

**Fig. 3:**
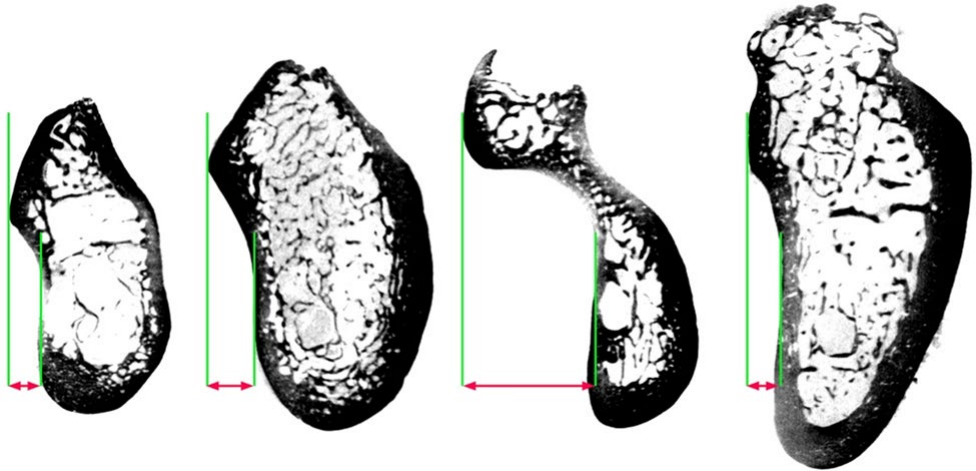
One example of each tooth region displaying the high variability of the ML width; PM, M1, M2, M3 from the left to the right. The red arrows indicate the width of the ML.

Surgical procedures in the posterior mandible, such as immediate or delayed implant installation, have the risk of violating the mandibular nerve or LPP [6, 19, 20]. In addition, LPP might cause injury of the sublingual artery, which in turn can lead to obstruction of the airways due to bleeding and hemorrhage and thereby to a serious life-threatening condition. The ML, which runs in this area of the mandible, may vary in its shape, width, and bony microarchitecture, which in turn might affect the risk of LPP and its consequences. The present anatomical study aimed to evaluate in the absence of teeth the variability of the ML, the microarchitecture of the adjacent bone, and whether the variable prominence/width of the ML is associated with the quality of the adjacent bone. So far, an undercut/prominent ML in the edentulous posterior mandible has been associated with a higher risk of LPP [20], however, ML width and the adjacent bone quality have not yet been precisely measured. The results of the present study, which are based on µCT data, indicated that the ML becomes more prominent from the PM towards M2 region, and then decreases in its width again in the region of M3. The median ML width in the M2 region was 3.4 mm, however, it was highly variable in this region. While the minimum value was comparable to the other tooth regions, i.e., about 0.4 mm, the maximum ML width value in the M2 region exceeded 10 mm (Fig. 3). In comparison, the ranges of the ML width in the PM and M1 region were 0.4 to 2.9 mm and 0.4 to 6.7 mm, respectively. Further, the median ML width of the M2 region was almost 2-times higher compared to the M1 region, i.e., 3.4 versus 1.9 mm, and more than 2-times higher compared to the PM region, i.e., 1.6 mm. Since the M1 and M2 regions are one of the most frequent regions for implant installation, these results underline the need for an individual and accurate diagnostic prior to any surgical intervention, especially in the highly variable M2 region. These results are also supported by previous radiographic studies based on CBCT scans, which measured the depth of the lingual fossa in the presence and absence of teeth using the ML as a reference for the most prominent upper point of the lingual plate [21–26]. More specifically, in the presence of M1, the horizontal distance between the deepest point of the lingual fossa and the ML ranged from 0.5 to 5.1 mm [26], whereas in the absence of M1 this distance reached up to 10 mm [25]; when both M1 and M2 were present, the mean distance ranged from 1.4 to 2.2 mm [22–24]. Although these studies applied slightly different measurement methods, they underline well together with the present data the high variability of the ML width in the posterior region [22, 25].

In this context not only the shape, but also the lingual CtTh is a relevant parameter affecting the risk of LPP. The lingual CtTh has been previously investigated in both, dentate and edentulous posterior mandibles [14, 15, 27]. The mean CtTh of the lingual plate in dentate posterior mandibles varied between 0.9 and 1.6 mm [14], whereas the median lingual CtTh in edentulous PM and anterior molar areas was 1.7 and 1.6 mm, respectively. In addition, the lingual CtTh increased with higher alveolar ridge resorption up to median values of 2.4 mm [15]. The results presented herein are overall well comparable. Specifically, in the sub-mylohyoid region (i.e., region A) the CtTh slightly differed among the 4 tooth regions, with the highest median CtTh of 2 mm in the PM and M1 region and the lowest in the M3 region (1.6 mm). Similar results were found for the CtTh in the supra-mylohyoid region (i.e., region B) with median CtTh values ranging from 1.8 to 2.2 mm and, overall, the CtTh either in region A or B ranged from approximately 1 to 4.2 mm. However, despite the highly variable ML width, the CtTh values appeared to remain unchanged, as neither simple correlations in the different tooth regions nor the generalized linear model showed any indication for an association between the ML width and the CtTh. Beside the quantity/thickness also the quality of the adjacent cortical bone was measured herein, i.e., the Avg.Ct.BV/TV. However, except for a higher range of the values in the more distal aspects (i.e., in the M2 and M3 regions), neither any differences in the Avg.Ct.BV/TV among the different tooth regions, nor any clinically relevant correlations and associations could be detected. Specifically, only in the M2 region a higher Avg.Ct.BV/TV correlated with a lower ML width, but when taking into account the multilevel nature of the data, no association between the Avg.Ct.BV/TV and ML width could be observed.

For long-term stability of dental implants, the quality of the trabecular bone is of crucial importance. In the maxilla, the quality of the trabecular bone of different implant anchorage sites (i.e., anterior, posterior, and zygomatic region) has been assessed by µCT [28], however, only a limited number of studies raised this question for the edentulous posterior mandible [15, 29–31]. In the present study the Avg.Tb.BV/TV was calculated for the trabecular bone adjacent to the lingual cortex and mylohyoid line, but not for the whole trabecular compartment. A previous histomorphometric study on the trabecular bone quality in the edentulous mandible has shown for the whole trabecular compartment median values of 22.3 and 18.6% in the PM and anterior molar region, respectively [15]. Comparable values have been reported for dentate specimens based on µCT analysis, i.e., a mean trabecular BV/TV of 18.5% in the posterior mandible [31], while also higher mean BV/TV values of 34.4% have been reported for the posterior edentulous mandible measured in the entire trabecular compartment [30]. In comparison, herein the median values of Avg.Tb.BV/TV of the A and B region ranged across the four tooth regions from 13.2 to 17.9% and from 16.7 to 21.9%, respectively. The different ROIs and often low number of specimens for µCT analysis may – at least partly – explain these discrepancies in the BV/TV values. Further, in the present study, the lowest median value of 13.2% was observed in the submylohyoid (A) region of M3, which was also significantly lower compared to the PM and M1 region. However, while Avg.Tb.BV/TV correlated significantly positive with CtTh in the PM and M1 region, no relevant association between the Avg.Tb.BV/TV and the ML width could be observed.

The present study has several limitations, such as a limited sample size and missing information on the timepoint of tooth loss and/or wear of dentures. Further, any information on the cause of death and medication intake, especially the intake of medication influencing bone remodeling, was lacking. The cross-sectional character also does not allow to draw any conclusions on changes of the ML width due to aging or increased time after tooth loss. Finally, all specimens were aged > 60 years, which make the results not representable/applicable for a younger population. In this context also the applied methods should be discussed critically. The specimens used herein were partially or fully edentulous, which did not allow an exact orientation according to the occlusal plane. Instead, it was decided to vertically orient the scans based on the PM region, which resembled well the natural position of the mandible. Nevertheless, a certain bias in the orientation might remain, which in turn might affect the exact measurement of the ML width. However, as the orientation of all samples has been performed in the same way minor changes in the actual ML width most likely do not affect the assessment of any relation and/or correlation to the bone microarchitecture parameters of the neighboring bone. Further, the use of µCT data has advantages but also some disadvantages compared to histomorphometry or CBCT. On a positive side, previous studies confirmed µCT as a similar reliable technique as histomorphometry to assess bone microarchitecture [32, 33]. In addition, µCT allowed herein with a single scan to combine the information of several cross-sectional images within a tooth region, which would be rather labor- and cost-intensive with conventional histomorphometry; this resembled a major advantage of µCT. On a negative side, CBCT would allow to collect more easily the data from a higher number of cases, yet CBCT still has limitations regarding detailed assessment of bone microarchitecture, which was one of the main aims herein [30]. Hence, altogether µCT appeared as a proper method for the specific aim of the present study.

## Conclusion

According to the results of the present anatomical study, and with respect to all limitations, one can conclude that the lingual shape of the edentulous alveolar ridge, i.e., the shape of the ML width, was not associated with the cortical and trabecular bone quality in the adjacent bone, while gender and tooth region had a significant effect. Specifically, the ML width was lower in female specimens, but peaked in the M2 region with a median width of 3 to 4 mm. In addition, it was confirmed that the ML is in general a highly variable structure, especially in the M2 region, which underlines the need for an individual and accurate diagnostic prior to any surgical intervention.

### Electronic supplementary material

Below is the link to the electronic supplementary material.

Supplementary Material 1

## Data Availability

The datasets used and/or analysed during the current study available from the corresponding author on reasonable request.
