# Supplementary material for: The mylohyoid line is highly variable but does not affect the microarchitecture of the edentulous alveolar bone – an anatomical micro-CT study
Source: BMC Oral Health. 2024 May 3;24:528. doi: 10.1186/s12903-024-04293-8 (PMC11069156; doi:10.1186/s12903-024-04293-8)
Supplement: Supplementary file 1 — Supplementary Material 1 [file 12903_2024_4293_MOESM1_ESM.pdf]

ethik kommission
Medizinische Universität Wien

Borschkegasse 8b/6
1090 Wien, Österreich
T +43(0)1 404 00-21470, 22440
F +43(0)1 404 00-16900
ethik-kom@meduniwien.ac.at
http://ethikkommission.meduniwien.ac.at/

Votum:

EK Nr: 1933/2020
Projekttitel: Histomorphometrische Untersuchung der Linea mylohyoidea mittels µCT
Antragsteller/in: Frau Julia Kappenberger
Institution: Universitätszahnklinik Wien, Fachbereich Orale Chirurgie, Institut für Anatomie und Zellbiologie
Sponsor: Medizinische Universität Wien

Teilnehmende Prüfzentren:

**Ethik-Kommission**	**Prüfzentrum**	**Prüfärztin/arzt**
Ethikkommission der Medizinischen Universität Wien	MUW / Universitätszahnklinik Wien - Fachbereich für orale Chirurgie	Herr Dr med. dent. Danijel Domic

Die Stellungnahme der Ethik-Kommission erfolgt aufgrund folgender eingereichter Unterlagen:

Lebenslauf (CV)

**Name**	**Version**	**Datum**
CV Danijel Domic	V2	05.09.2019
CV Prof. Ulm	V2	25.09.2019

Sonstige

**Name**	**Version**	**Datum**
Verpflichtungserklaerung Julia Kappenberger	V1	15.07.2020
unterschriebenes-Antragsformulare	V1	08.10.2020

Studienprotokoll (Prüfplan)

**Name**	**Version**	**Datum**
Studienprotokoll-JuliaKappenberger_V2	V2	20.10.2020

Die Kommission fasst folgenden Beschluss (mit X markiert):

☒	Es besteht kein Einwand gegen die Durchführung der Studie.<br><br>ACHTUNG: Unter Berücksichtigung der "ICH-Guideline for Good Clinical Practice" gilt dieser Beschluss ein Jahr ab Datum der Ausstellung. Gegebenenfalls hat der Antragsteller eine Verlängerung der Gültigkeit rechtzeitig zu beantragen.

Ergänzende Kommentare der Sitzung am 13.10.2020:

Zum Prüfplan:
Im Abschnitt "Datenschutz" ist der Begriff "pseudoanonymisiert" durch "pseudonymisiert" zu ersetzen.
Es ist zu ergänzen, wo die Excel-Datei mit den erhobenen Daten gespeichert wird und wer darauf Zugriff hat (es muss sich um einen Server der MedUni Wien handeln und die Daten müssen für die MedUni Wien/die am Projekt Beteiligten weiter zur Verfügung stehen).

Zum Antrag:
Die Angabe der Klinikzugehörigkeit von Frau Dr. Hirtler ist im Reiter Zentren zu korrigieren (Institut für Anatomie und Zellbiologie).

Die Ethik-Kommission ersucht die Antragsteller, bei der Wiedervorlage von geänderten Unterlagen ein Exemplar mit hervorgehobenen Änderungen beizulegen.

Ergänzende Kommentare:

Nachtrag vom 21. Oktober 2020:
Die Antragsteller legen am 20.10.2020 überarbeitete Unterlagen vor, die von der Ethik-Kommission akzeptiert werden.

Die aktuelle Mitgliederliste der Ethik-Kommission ist unter folgender Adresse abrufbar:
http://ethikkommission.meduniwien.ac.at/ethik-kommission/mitglieder/

Mitglieder der Ethik-Kommission, die für diesen Tagesordnungspunkt als befangen anzusehen waren und daher laut Geschäftsordnung an der Entscheidungsfindung/Abstimmung nicht teilgenommen haben: **keine**

Dieses Dokument ist für berechtigte Benutzer/innen in digitaler Form unter folgender Adresse abrufbar:
https://ekmeduniwien.at/vote/20817/download/

	**Unterzeichner**	Dr. Jürgen Zezula
	**Datum/Zeit-UTC**	2020-10-21T21:41:15Z
	**Prüfinformation**	Informationen zur Prüfung der elektronischen Signatur finden Sie unter: https://www.signaturpruefung.gv.at
